# Supplementary material for: Crosstalk between regulatory elements in disordered TRPV4 N-terminus modulates lipid-dependent channel activity
Source: Nat Commun. 2023 Jul 13;14:4165. doi: 10.1038/s41467-023-39808-4 (PMC10344929; doi:10.1038/s41467-023-39808-4)
Supplement: Supplementary file 1 — Supplementary Information [file 41467_2023_39808_MOESM1_ESM.pdf]

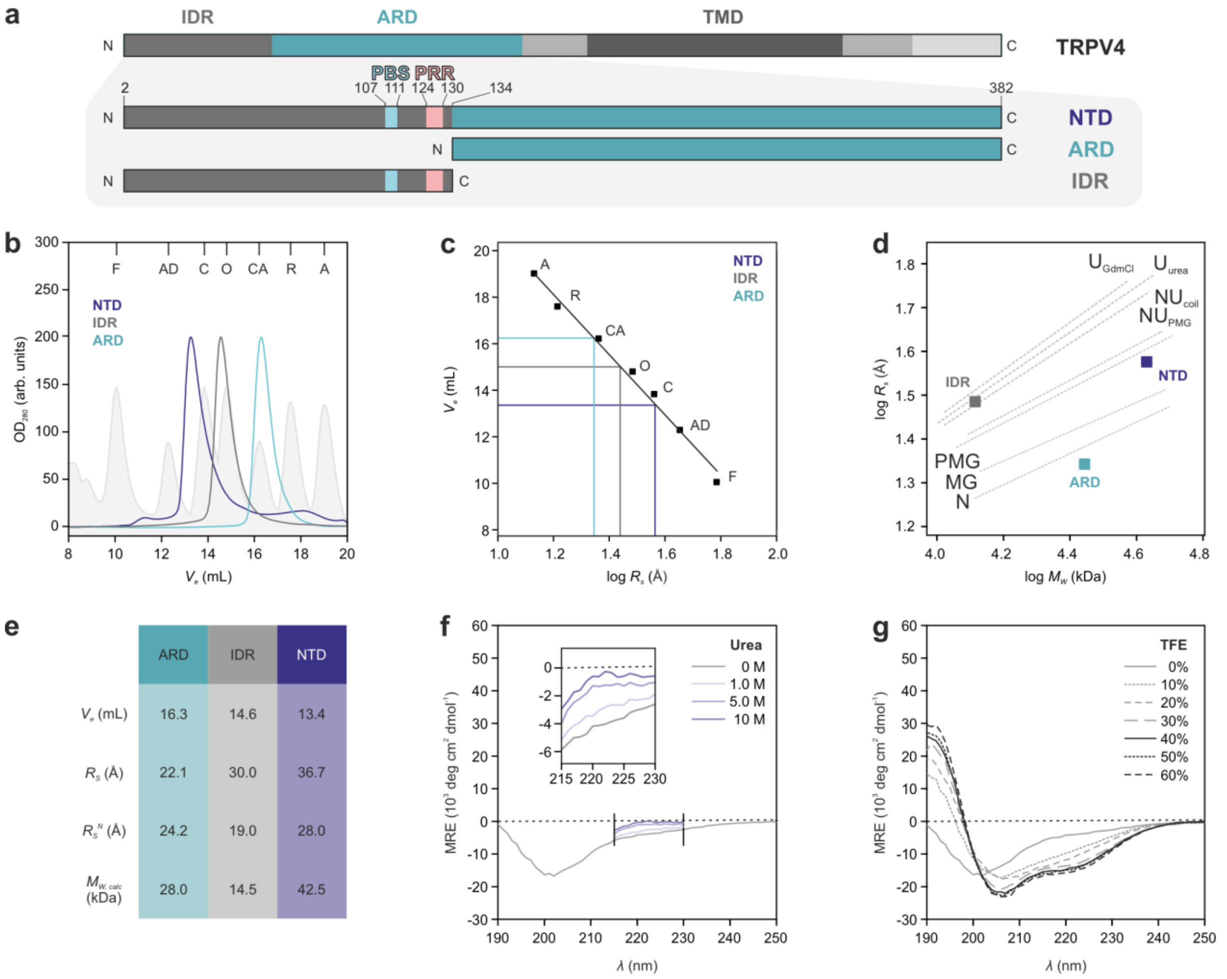

Supplementary Figure 1: Structural characterization of heterologously expressed TRPV4 N-terminal constructs.
a Construct design for *G. gallus* TRPV4 NTD, ARD and IDR. Important regulatory sites (PBS: PIP_2 binding site; PRR: proline rich region) are highlighted in light blue and light pink, respectively.
b Analytical size-exclusion chromatography (SEC) of TRPV4 NTD, IDR and ARD. SEC profiles of standard proteins Stokes radius (R_S) determination are shown as filled curves (light grey): F: ferritin; AD: alcohol dehydrogenase; C: conalbumin; O: ovalbumin; CA: carbonic anhydrase; R: ribonuclease A; A: aprotinin. SEC profiles of NTD, IDR and ARD were normalized to an optical density at 280 nm (OD_{280}) of 200 arb. units (arbitrary units).
c Calibration curve (black) generated with elution volumes of protein standards shown in (b).
d Structural analysis of TRPV4 N-terminal constructs based on M_w and determined R_S. The ARD can be generally classified as more-compact native-like protein, the IDR as a native coil-like protein and the NTD as a pre-molten globule-like protein, i.e. a mixture of unfolded and globular subdomains. (N: native; MG: molten globule; PMG: pre-molten globule; NU_{PMG}: native pre-molten globule like; NU_{coil}: native coil-like; U_{urea}: urea unfolded; U_{GdHCl}: guanidinium hydrochloride unfolded[1]).
e Stokes radii (R_S) calculated from SEC elution volumes (V_e) in calibration curve in (d). Theoretical R_S values (R_S^N) assuming globular, monomeric proteins under native conditions were determined using described procedures[1] based on the calculated molecular weights ($M_{W,\,calc}$) of the three constructs.
f, g Far-UV circular dichroism (CD) spectra of purified IDR with increasing amounts of trifluoroethanol (TFE) and urea demonstrate that secondary structure can only be induced at high amounts of TFE and no secondary structure content is lost by urea addition, thereby classifying the IDR as truly intrinsically disordered. Due to high detector voltages in the presence of high urea concentrations, only wavelengths above 215 nm were recorded. The relevant region for changes in secondary structure content is shown in the box. Spectra were plotted as the mean residue ellipticity (MRE) versus the wavelength λ.
Source data are provided as a Source Data file.

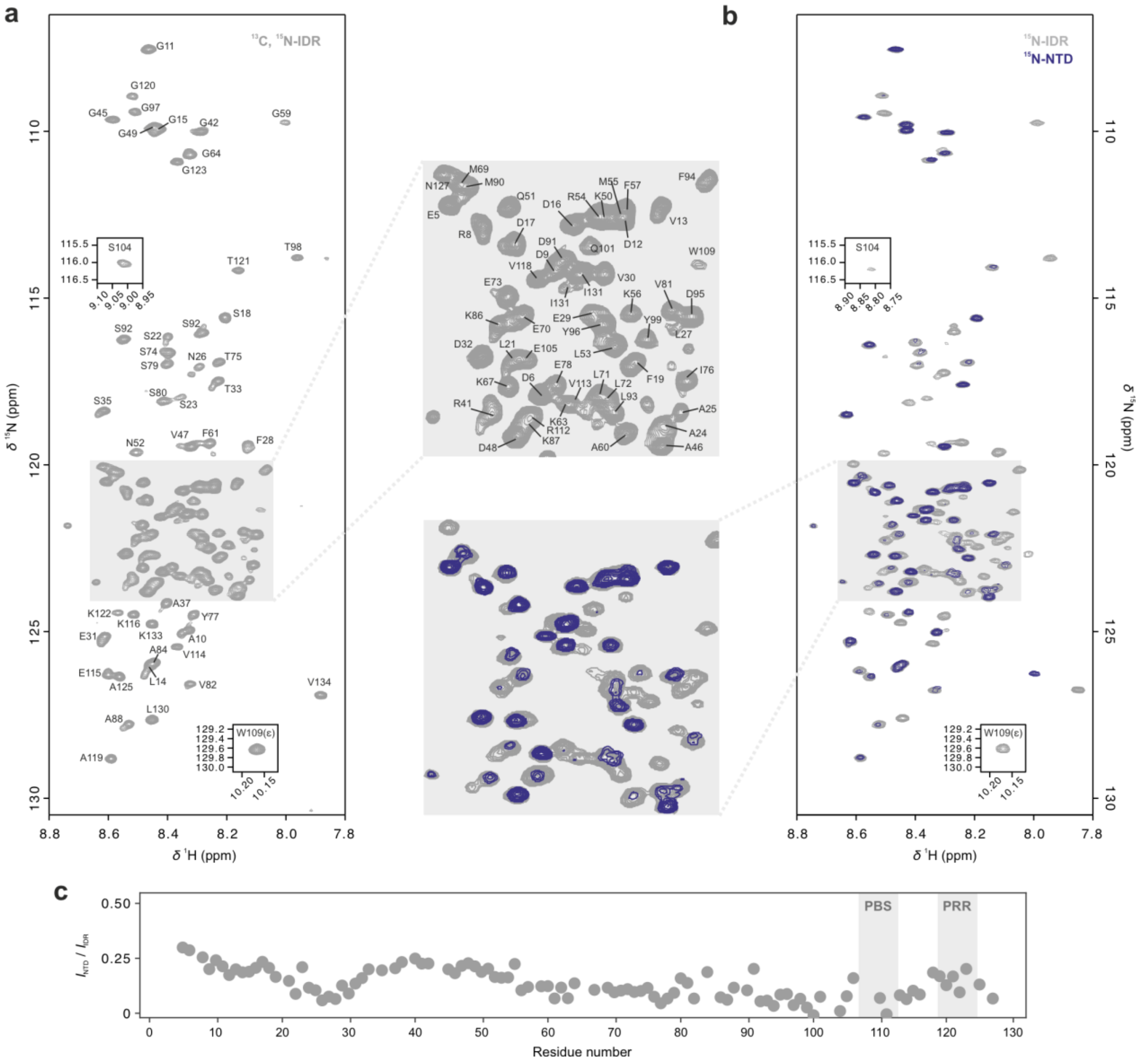

Supplementary Figure 2: [^{1}H, ^{15}N]-TROSY-HSQC NMR spectrum of TRPV4 IDR and comparison with NTD.
a Backbone amide resonance assignments of native *G. gallus* TRPV4 IDR comprising residues 2-134[2].
b Overlay of [^{1}H, ^{15}N]-TROSY-HSQC spectra of ^{15}N-labeled IDR (grey) and NTD (blue).
c Relative peak intensity of residues within the IDR in isolation or in the context of the NTD, i.e. in the presence of the ARD. Reduced signal intensity, i.e. a low I_{NTD}/I_{IDR} ratio, suggesting contact sites between IDR and ARD. Previously described functionally important sites in the C-terminal IDR are highlighted by grey boxes (PBS: PIP_2 binding site; PRR: proline rich region).
Source data are provided as a Source Data file.

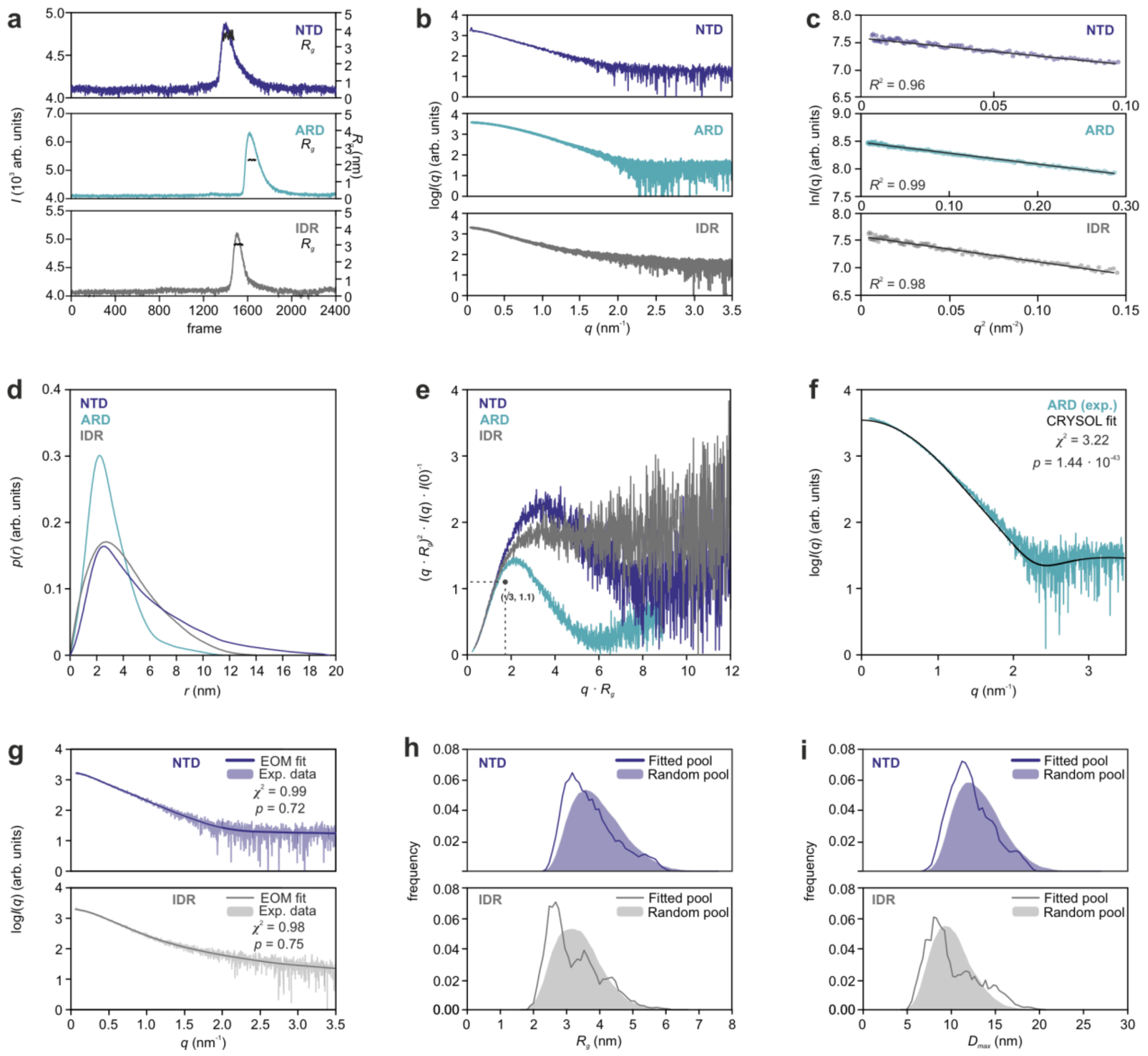

Supplementary Figure 3: SEC-SAXS and Ensemble optimization method (EOM) analysis of TRPV4 N-terminus.

a SEC-SAXS profiles of NTD (blue), ARD (cyan), and IDR (grey). Plotted are the partially integrated scattering intensities, I (in arbitrary units), from sequentially recorded 1D-scattering data frames measured throughout the SEC-elution of each sample. After background/buffer subtraction, the respective radii of gyration (R_g) correlation through the elution peaks of each sample were calculated using the Guinier approximation (indicated in black, with the magnitude on the right axis).

b X-ray scattering profiles of NTD (blue), ARD (cyan), and IDR (grey) plotted as the logarithm of the scattering intensity log$I(q)$ (in arbitrary units) versus the momentum transfer, q.

c Guinier-plots (ln$I(q)$ in arbitrary units, vs q^2, plotted to low-angle: $qR_g < 1.3$) of NTD (blue), ARD (cyan), and IDR (grey).

d Real-space pair-distance distribution functions, or $p(r)$ profiles (in arbitrary units) calculated for NTD (blue), ARD (cyan), and IDR (grey). $p(r)$ functions were scaled to an area under the curve value of 1.

e Dimensionless Kratky plot of NTD (blue), ARD (cyan), and IDR (grey). The scattering from the ARD is consistent with a compact/globular particle, while the IDR is highly flexible. The NTD scattering data shows a combination of these features. The maximum at $qR_g = \sqrt{3}$ and $(qR_g)I(q)I(0)^{-1} = 1.1$ representative of an ideally globular protein is indicated.

f CRYSOL fit[3] of isolated ARD shows an unsatisfactory match between experimental and predicted scattering curves based on the ARD X-ray structure (PDB: 3W9G) as demonstrated by a χ^2 value of 3.22 and a CorMap p value of $1.44 \cdot 10^{-43}$.[4] Data are plotted as log$I(q)$ (in arbitrary units) vs. q. The statistical analyses of the data-model fit were carried out using the reduced χ^2 method (one-tailed distribution)[5] and CorMap[4] (one-tail Schilling distribution) test methods. The determined χ^2 and CorMap p values are indicated in (g).

g Fit of EOM refined IDR and NTD volume-fraction weighted model ensembles (solid line) to the experimental SAXS data of NTD (dark blue) and IDR (grey). Data are plotted as log*I*(*q*) (in arbitrary units) vs. *q*. The statistical analyses of the fits were carried out using the reduced χ^2 method[5] (one-tailed distribution) and CorMap (one-tail Schilling distribution) test methods. The determined χ^2 and CorMap[4] *p* values are indicated in the corresponding graphs.
h, i R_g and D_{max} distributions of the random pool of generated IDR and NTD structures (filled curves) compared to the R_g and D_{max} distributions of the EOM refined ensembles (solid lines).
Source data are provided as a Source Data file.

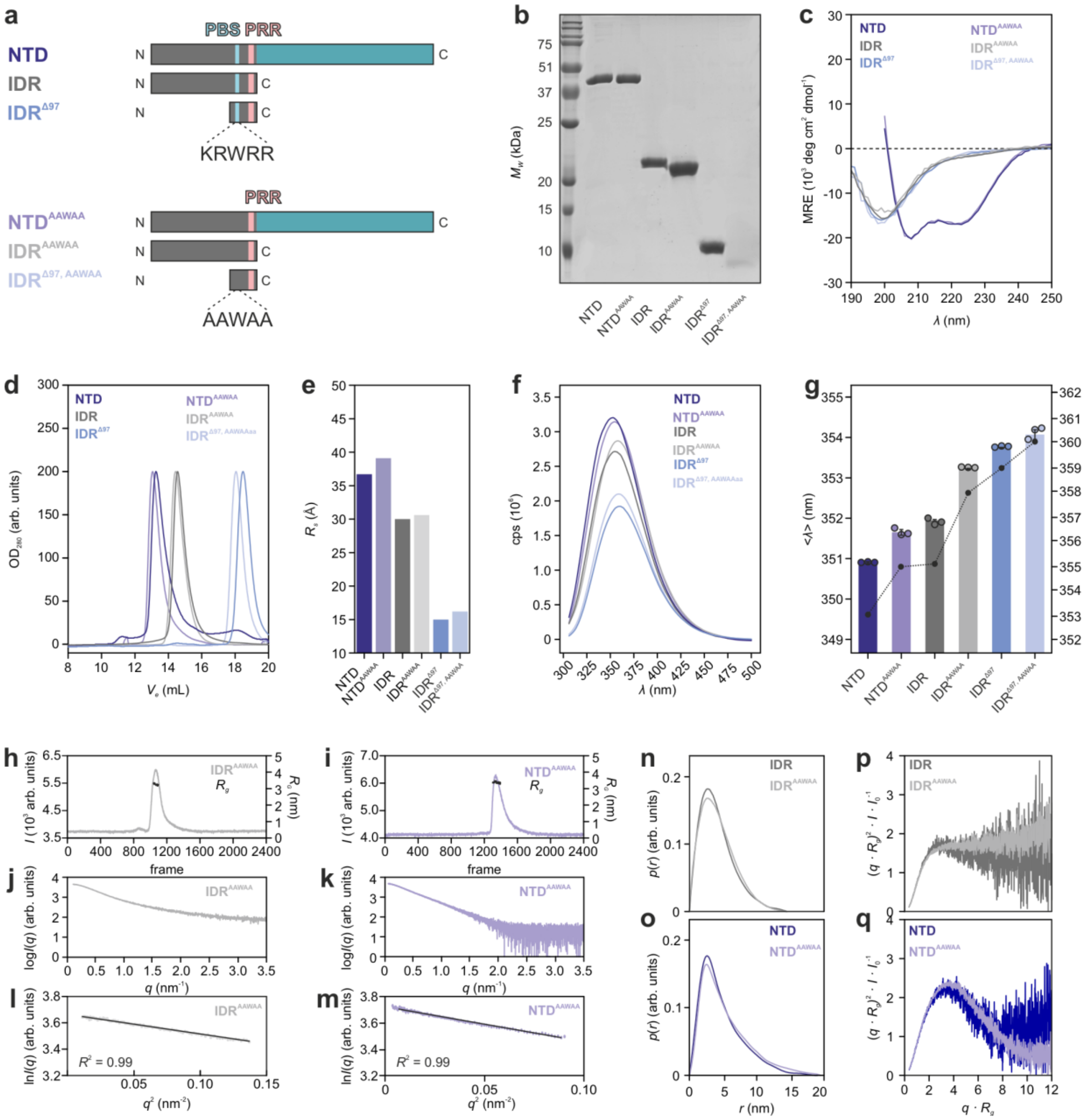

Supplementary Figure 4: PIP_2-binding site mutation does not affect structural integrity of TRPV4 N-terminal constructs.

a Topology of native and PIP_2 binding site mutant constructs, NTD, IDR, IDR$^{\Delta N97}$. In the AAWAA mutants, the PIP_2-binding site ^{107}KRWRR^{111} (PBS, yellow) is exchanged to ^{107}AAWAA^{111} (dark grey). ARD, cyan; IDR, light grey; proline rich region (PRR), red.

b Coomassie-stained SDS-PAGE of purified native and AAWAA mutant constructs. M_W is the molecular weight (in kDa). SDS-PAGE analysis was carried out once with all constructs side by side to demonstrate sample purity and relative size.

c, d Far-UV CD spectra and SEC profiles of purified constructs. MRE is the mean residue ellipticity and OD_{280} is the optical density at 280 nm (in arbitrary units).

e Stokes radii (R_S) of native and AAWAA mutant constructs estimated from the elution volumes observed in (d).

f, g Tryptophan fluorescence spectroscopy of native and corresponding AAWAA mutant constructs. Bars show intensity-weighted average wavelength <λ> from 320 to 380 nm (left axis). Data in g are presented as the mean value +/- SEM from n=3. Fluorescence emission maxima λ_{max} are shown as black circles connected by dotted lines (right axis). Fluorescence intensity is presented in counts per second (cps).

h, i SEC-SAXS profiles of IDR^{AAWAA} (h) and NTD^{AAWAA} (i). Plotted are the partially integrated scattering intensities, I, from sequentially recorded 1D-scattering data frames measured throughout the SEC-elution of each sample. After background/buffer subtraction, the respective radii of gyration (R_g) correlation through the elution peaks

of each sample were calculated using the Guinier approximation and are indicated in black (with the magnitude on the right axis).

j, k X-ray scattering profiles of IDR^{AAWAA} (j) and NTD^{AAWAA} (k) plotted as the logarithm of the scattering intensity log(I(q)) (arbitrary units) versus the momentum transfer, q.

l, m Guinier-plots (ln$I$(q) vs q^2, plotted to low-angle: qR_g < 1.3) of IDR^{AAWAA} (l) and NTD^{AAWAA} (m).

n, o Dimensionless Kratky plots of IDR and IDR^{AAWAA} (n) as well as NTD and NTD^{AAWAA} (o).

Source data are provided as a Source Data file.

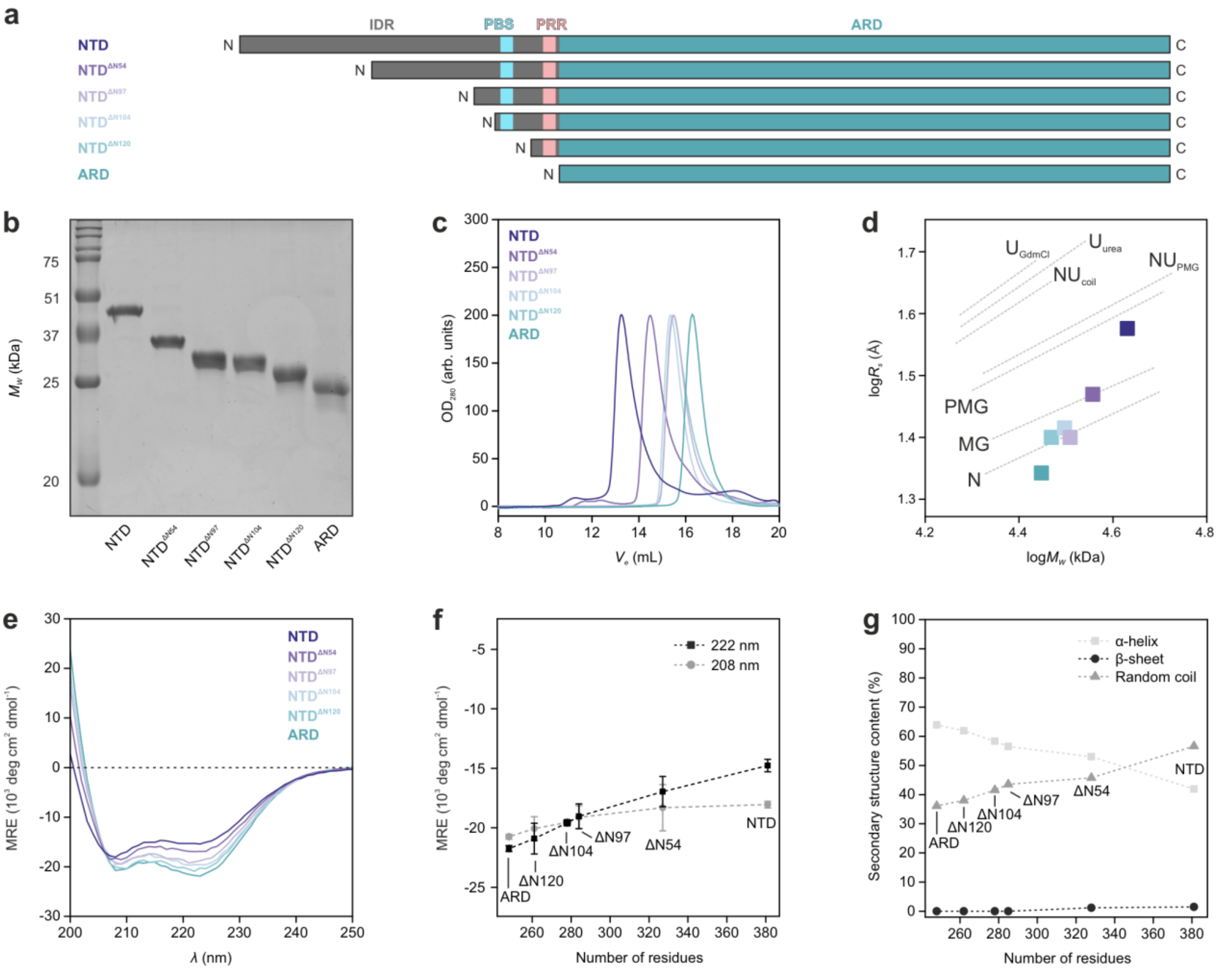

Supplementary Figure 5: Structural characterization of N-terminal TRPV4 NTD deletion mutants.

a N-terminally truncated TRPV4 NTD constructs used in this study.

b, c Coomassie-stained SDS-PAGE and SEC profiles of NTD deletion constructs. M_W is the molecular weight (in kDa) and OD_{280} is the optical density at 280 nm (in arbitrary units). SDS-PAGE analysis in (b) was done once to demonstrate sample purity and expected molecular weight.

d R_s-M_W analysis of NTD deletion constructs. NTD: V_e = 13.4 mL, R_s = 36.7 Å, M_W = 42.5 kDa; $NTD^{\Delta N54}$: V_e = 14.6 mL, R_s = 29.6 Å, M_W = 37.0 kDa; $NTD^{\Delta N97}$: V_e = 15.4 mL, R_s = 25.4 Å, M_W = 32.3 kDa; $NTD^{\Delta N104}$: V_e = 15.4 mL, R_s = 26.0 Å, M_W = 31.4 kDa; $NTD^{\Delta N120}$: V_e = 15.5 mL, R_s = 25.5 Å, M_W = 29.4 kDa; ARD: V_e = 16.3 mL, R_s = 22.1 Å, M_W = 28.0 kDa. Dotted lines reflect empirical R_s-M_W scaling relationships for folded globular proteins (N), proteins in molten globule (MG) and pre-molten globule (PMG) states, proteins with pre-molten globule like (NU_{PMG}) and random coil behavior (NU_{coil}) under physiological conditions, and proteins under chemically denaturing conditions (U_{urea} for urea, U_{GdmCl} for guanidium hydrochloride)[1].

e, f Far-UV CD spectra of NTD deletion constructs and mean residue ellipticity (MRE) at 208 nm and 222 nm. Data are presented as the mean value +/- SEM from n=3 biological replicates.

g BeStSel[6] based secondary structure analysis plotted versus the number of amino acids in the NTD constructs shows that overall secondary structure content of the ARD is not affected by the consecutive deletion of the intrinsically disordered IDR.

Source data are provided as a Source Data file.

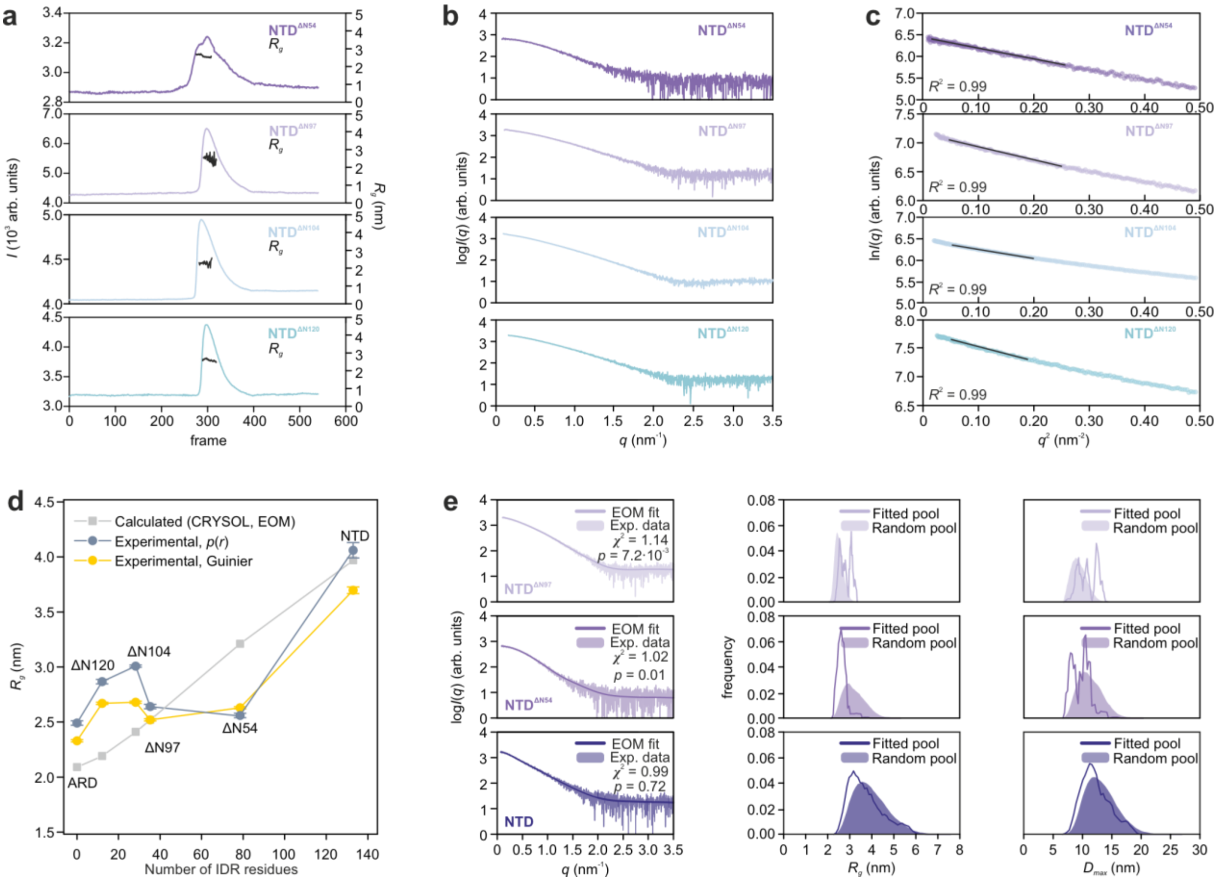

Supplementary Figure 6: SAXS analysis of N-terminal deletion mutants of TRPV4 NTD

a SEC-SAXS profiles of NTD$^{\Delta N54}$, NTD$^{\Delta N97}$, NTD$^{\Delta N104}$ and NTD$^{\Delta N120}$. Plotted are the partially integrated scattering intensities, *I* (in arbitrary units), from sequentially recorded 1D-scattering data frames measured throughout the SEC-elution of each sample. After background/buffer subtraction, the respective radius of gyration (R_g) correlation through the elution peaks of each sample were calculated using the Guinier approximation and are indicated in black (with the magnitude on the right axis).

b Background subtracted SAXS profiles of the NTD deletion mutations extracted from the SEC traces shown in (a), plotted as log$I(q)$ (in arbitrary units) vs. q.

c Corresponding Guinier-plot of scattering data shown in (b).

d Experimental and calculated radius of gyration (R_g) plotted versus the number of IDR residues included in the respective NTD constructs. The R_g of the isolated ARD was calculated with CRYSOL[3] (grey). The R_g of the NTD and the deletion mutants represent the average R_g value of a random pool of conformations calculated via EOM analysis[7] assuming a random chain behavior of the IDR. R_g values were experimentally obtained from the Guinier analysis (yellow) or the pair-distance distribution (blue-grey). Data are represented as mean values +/- Guinier fitting errors and the $p(r)$ fit.

e EOM analysis of NTD$^{\Delta N97}$, NTD$^{\Delta N54}$, and wildtype NTD. Shown is the EOM fit (solid line) to the respective experimental data log$I(q)$ (in arbitrary units) vs. q (left). On the right, the EOM derived R_g and D_{max} distributions comparing a random pool of generated conformations (filled curve) to the EOM-selected ensemble fit to the experimental data. The experimental SAXS profiles of the NTD$^{\Delta N104}$ and NTD$^{\Delta N120}$ mutants could not be reliably fitted to a random pool of conformations using the EOM algorithm (fits not shown). Data are plotted as log$I(q)$ (in arbitrary units) vs. q. The statistical analyses of the data-model fits were carried out using the reduced χ^2 method[5] (one-tailed distribution) and CorMap[4] (one-tail Schilling distribution) test methods. The determined χ^2 and CorMap p values are indicated in theSource data are provided as a Source Data file.

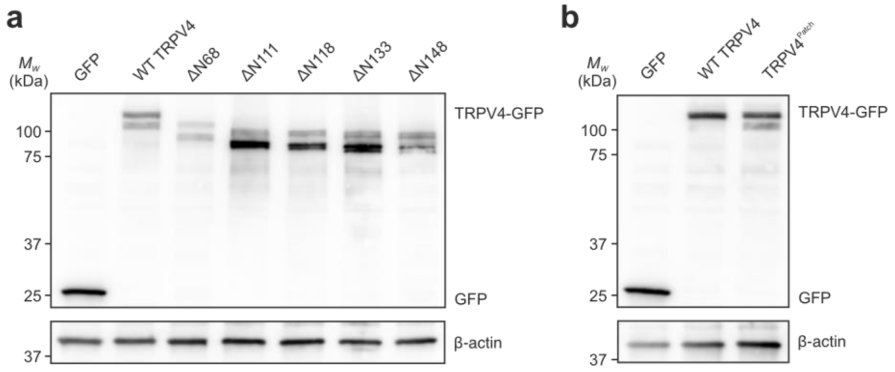

Supplementary Figure 7: Expression of TRPV4 N-terminal constructs in MN-1 cells.
a Western Blot of human TRPV4 N-terminal deletion constructs (n=2, biological replicates).
B Western Blot of human TRPV4^{Patch} mutant (n=1).
Source data are provided as a Source Data file.

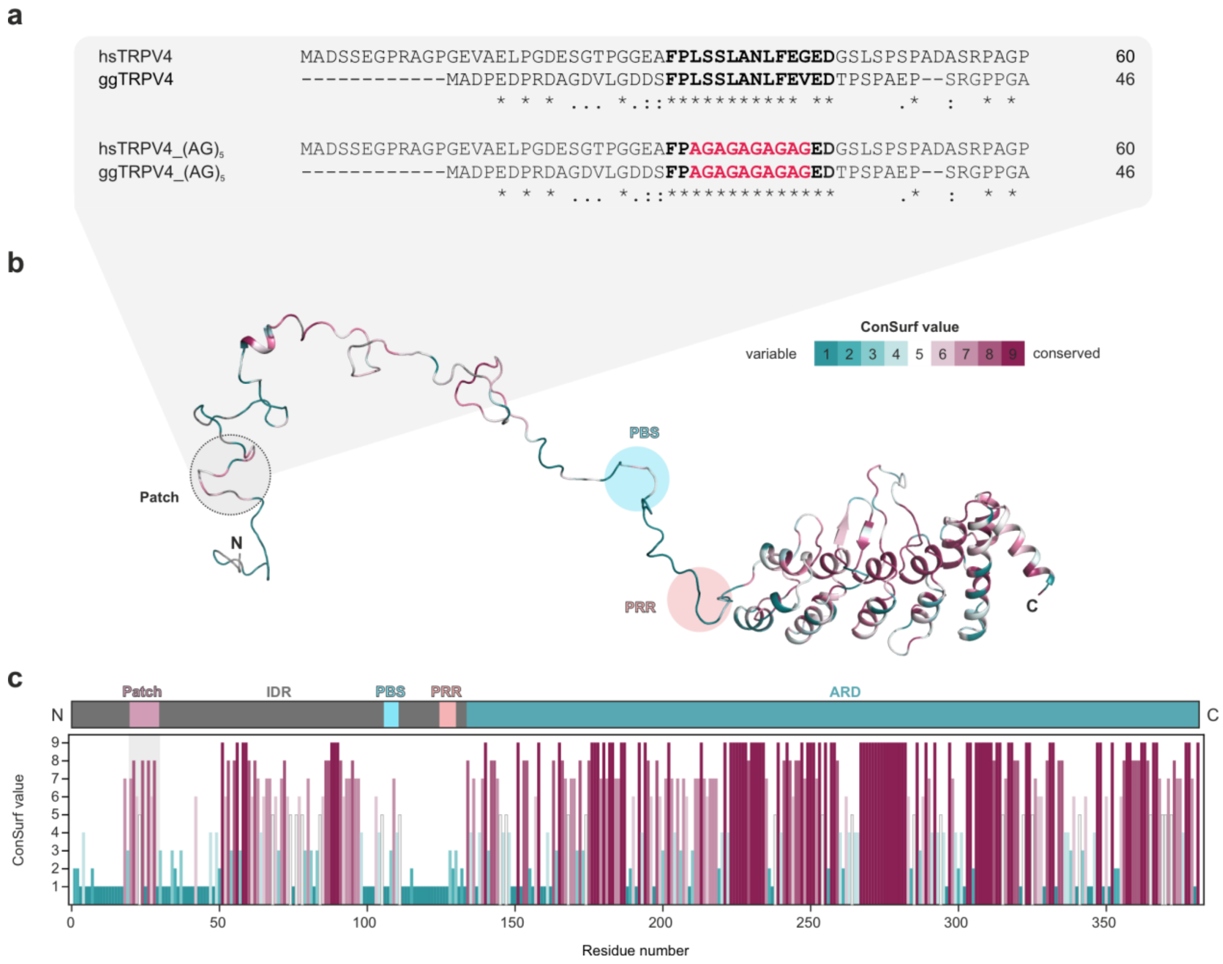

Supplementary Figure 8: Sequence alignment and conservation of human and chicken TRPV4 IDR.

a Sequence alignment of N-terminal IDR of human and chicken TRPV4. The conserved regulatory patch is shown in bold. The corresponding $(AG)_5$ mutants of the conserved patch are shown below and highlighted in red. Alignments were carried out with ClustalOmega[8].

b Conservation of residues in TRPV4 NTD shown mapped onto a *G. gallus* TRPV4 NTD conformer obtained from our EOM analysis (Fig. 1). Analysis was carried out with the ConSurf server[9] (PBS: PIP_2 binding site; PRR: proline rich region).

c TRPV4 NTD topology and sequence conservation of TRPV4 NTD residues based on the ConSurf analysis[9].

Source data are provided as a Source Data file.

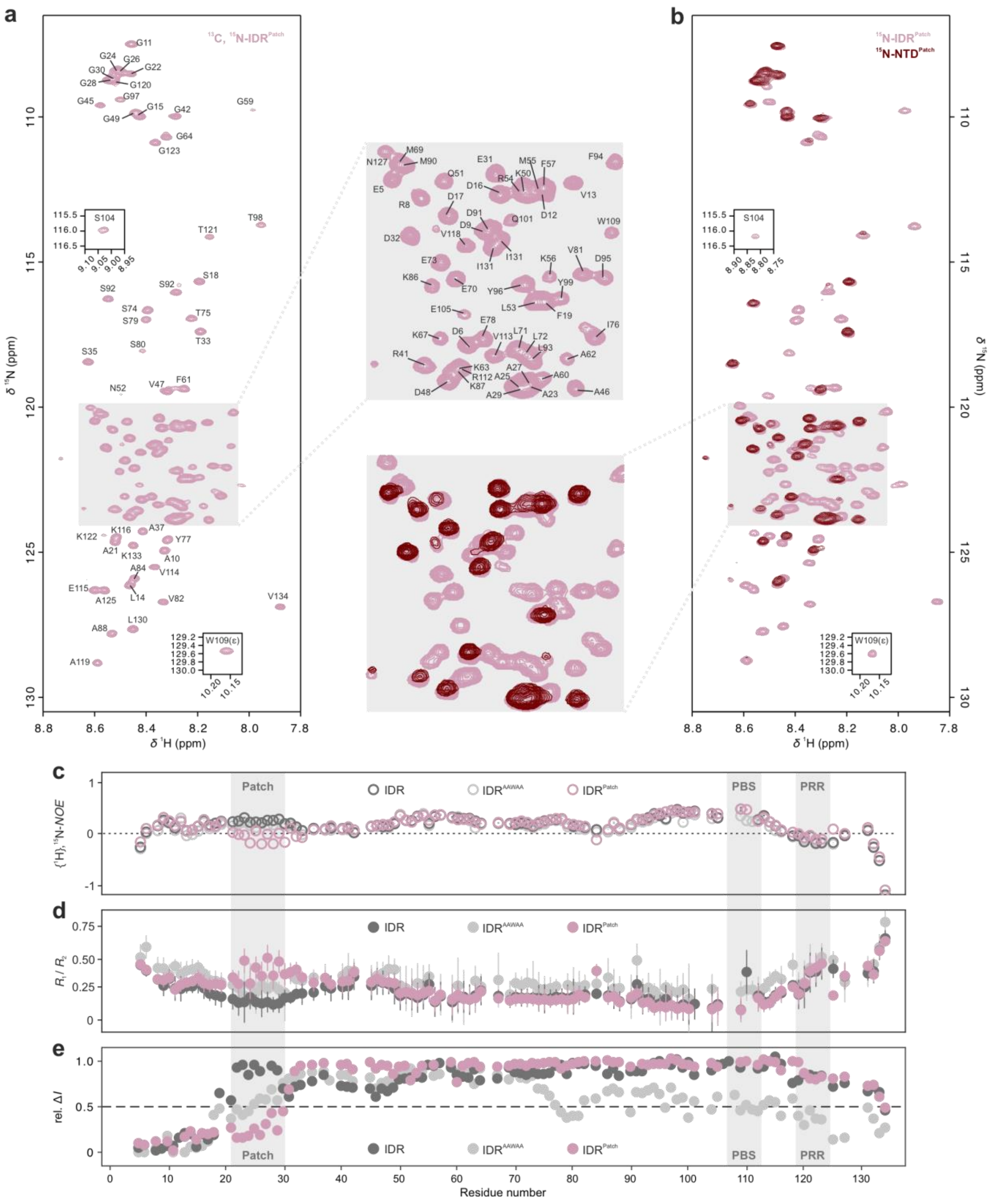

Supplementary Figure 9: [^{1}H, ^{15}N]-TROSY-HSQC NMR spectra of and backbone assignments of TRPV4 IDR^{Patch} and NTD^{Patch}.

a IDR with a mutated N-terminal patch, IDR^{Patch}. Residues ^{19}FPLSSLANLFEVE^{31} are exchanged to glycine and alanine yielding ^{19}FP(AG)$_5$E^{31}.

b Overlay of the HSQC spectra of ^{15}N-labeled IDR^{Patch} and NTD^{Patch}.

c, d {^{1}H},^{15}N-*NOE* and R_1/R_2 relaxation data of of ^{15}N-labeled IDR (dark grey), IDR^{AAWAA} (light grey), IDR^{Patch} (mauve). Data in (d) are presented as the mean value +/- the propagated errors of the R_1/R_2 ratios derived from the individual R_1 and R_2 fits.

e NMR signal intensity differences for ^{15}N-labeled IDR (dark grey), IDR^{AAWAA} (light grey) and IDR^{Patch} (mauve) in the absence and presence of POPC-POPG containing liposomes. For better comparison, the data from Fig. 6d, e for native IDR and IDR^{AAWAA} were added here.

Source data are provided as a Source Data file.

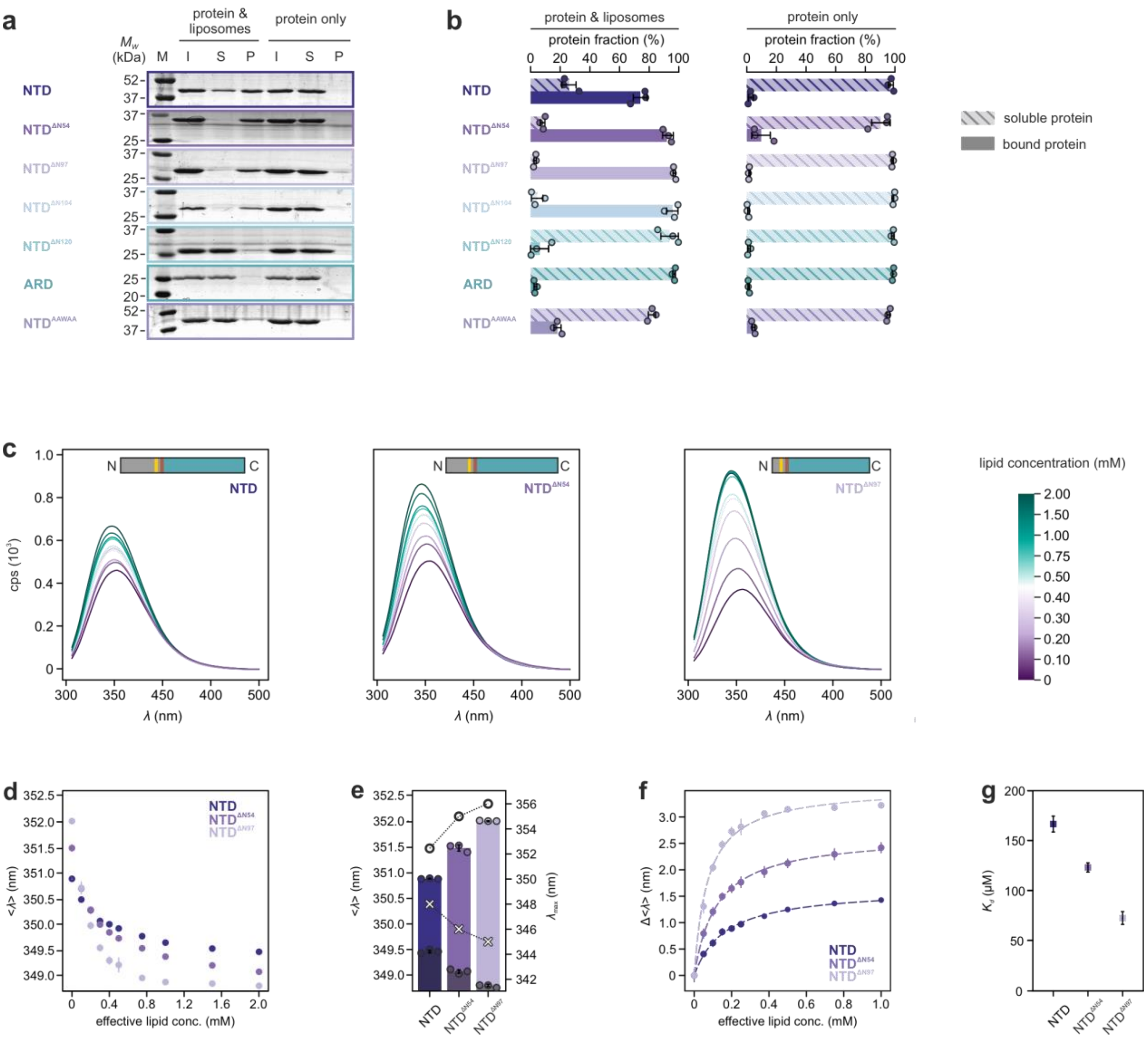

Supplementary Figure 10: Effect of N-terminal truncations on the lipid interaction of the TRPV4 NTD monitored via tryptophan fluorescence.

a Representative SDS-PAGE for liposome sedimentation assay. I: input; S: supernatant; P: pellet. Control experiments were carried out in the absence of liposomes (protein only). A lipid mixture of 50% (w/w) POPG and 50% (w/w) POPC was used to prepare liposomes with 100 nm diameter.

b Protein distribution between pellet or supernatant after centrifugation. The protein fraction in the pellet and supernatant was quantified via densitometry of SDS-PAGE protein bands using imageJ[10]. Data are presented as the mean value +/- SEM from n=3 biological replicates.

c Tryptophan fluorescence spectra of TRPV4 NTD and N-terminal deletion mutants in the presence of 0/0.1/0.2/0.3/0.4/0.5/0.75/1.0/1.5/2.0 mM lipids (100 nm liposomes, POPG:POPC in a 1:1 ratio).

d Intensity-weighted average wavelength <λ> plotted against the lipid concentration. Data are presented as mean values +/- SEM from n=3 biological replicates.

e Intensity-weighted average wavelength <λ> of TRPV4 NTD constructs without (colored bars) and in the presence of 2 mM lipid (grey bars) (<λ> on left axis). Tryptophan fluorescence emission maximum wavelengths λ_{max} without lipids and in the presence of liposomes (2 mM lipid) are shown as black circles and black crosses respectively (values for λ_{max} on right axis). Data are presented as mean values +/- SEM from n=3 biological replicates.

f Intensity-weighted average wavelength shift Δ<λ> plotted against the effective lipid concentration (half of total lipid concentration). The data were fitted with a Hill equation with a Hill coefficient of n=1 to obtain K_d values.

g Comparison of determined K_d values in (d) for NTD (165.1 ± 7.9 µM), NTD$^{\Delta N54}$ (122.1 ± 4.6 µM) and NTD$^{\Delta N97}$ (72.1 ± 6.4 µM). Data are presented as values +/- errors of the K_d fit. The error bars in (b, c, d) represent the SD of mean from n=3 technical replicates. Data are presented as mean values +/- The values and error bars in (g) are the fit values and fit errors from (f).

Source data are provided as a Source Data file.

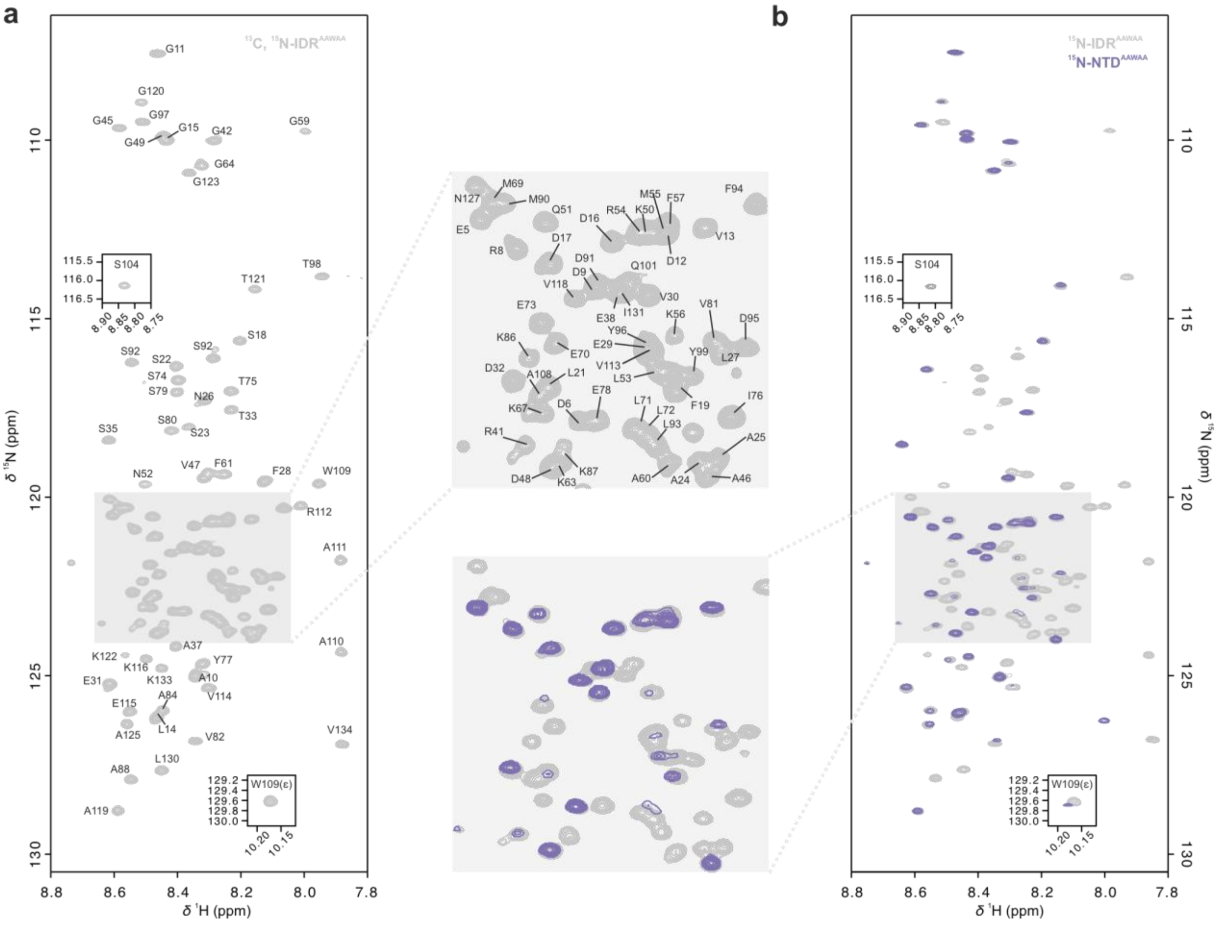

Supplementary Figure 11: [^{1}H, ^{15}N]-TROSY-HSQC NMR spectra of and backbone assignments of TRPV4 IDR^{AAWAA} and NTD^{AAWAA}.

a IDR with a mutated PIP$_2$ binding site, IDR^{AAWAA}, in which the basic residues in the stretch ^{107}KRWRR^{111} are mutated to alanine.

b Overlay of the spectra of ^{15}N-labeled IDR^{AAWAA} and NTD^{AAWAA}.

Source data are provided as a Source Data file.

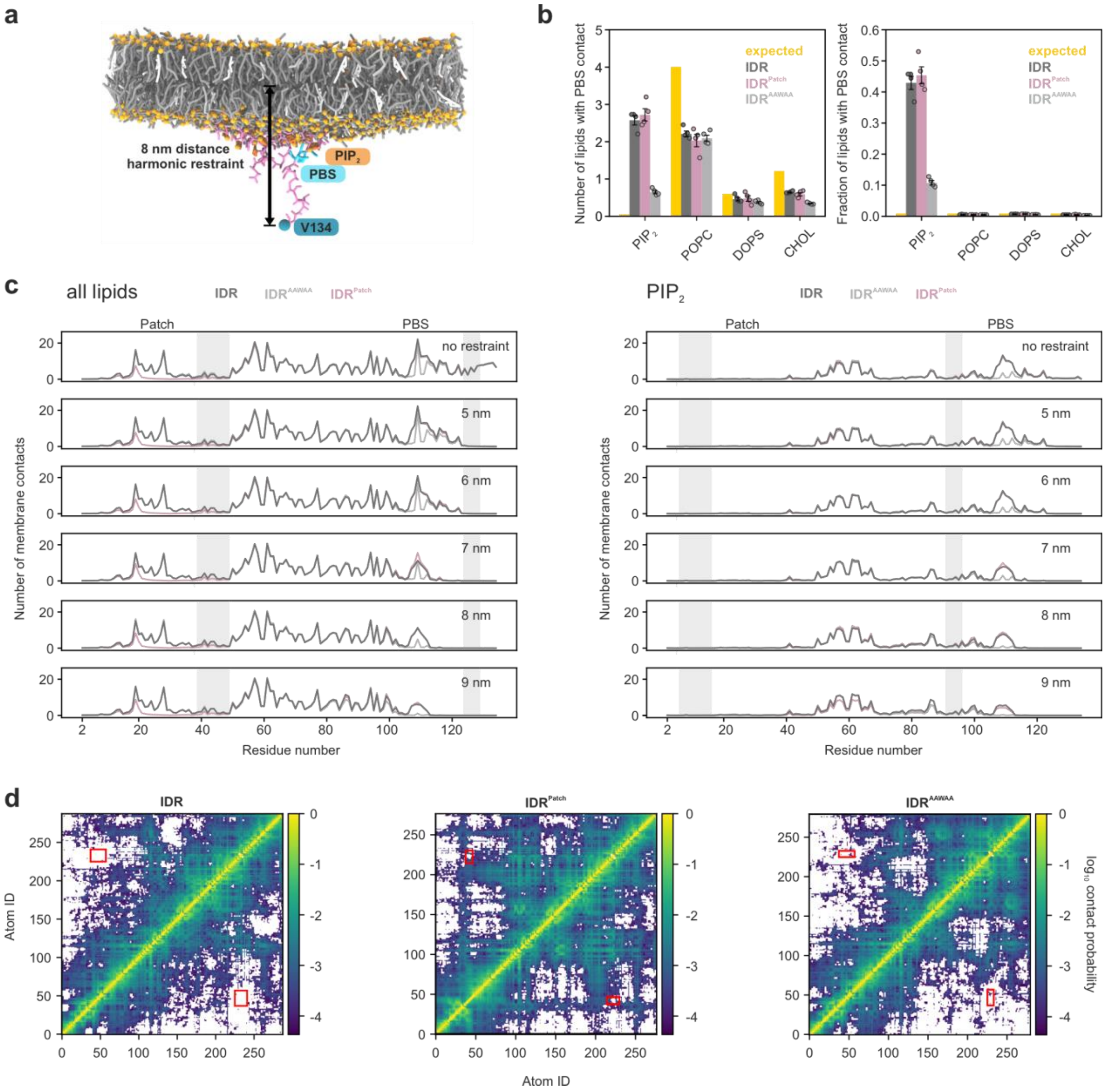

Supplementary Figure 12: TRPV4 IDR interactions with the plasma membrane through coarse-grained MD simulations.

a Snapshot of the bent membrane taken from one of the simulations of the native IDR (pink) with a height restraint of 8 nm (V134 (blue sphere) distance to membrane midplane) after 15.5 µs of simulation on a lipid bilayer membrane consisting of PIP_2 (1%, dark orange), POPC (69%, dark grey), DOPS (10 %, light gray) and cholesterol (20%, white) (see Supplementary Table 3). Headgroup phosphates are shown as orange spheres. The PIP_2-binding site of the TRPV4 IDR is highlighted in cyan.

b Absolute (left) and relative (right) number of contacts between the PIP_2-binding site (PBS) and each lipid type in simulations of the native IDR (dark grey), the patch mutant (light grey) and the IDR^{AAWAA} mutant (pink). Shown values are based on simulations carried out without a height restraint acting on V134. Grey bars indicate the number of expected contacts for the PIP_2-binding site solely based on the respective mol-fractions of lipid types in the simulated membrane. Data are presented as mean values +/- SEM from n=4 independent simulations.

c Average number of membrane contacts for each residue in the native IDR (dark grey), the IDR^{AAWAA} (light grey) and the IDR^{Patch} mutant (pink) on a lipid bilayer composed of POPC (69%), CHOL (20%), DOPS (10%), PIP_2 (1%). The location of the N-terminal patch and the PIP_2-binding site (PBS) are highlighted by grey boxes. On the left contacts for all lipids and on the right only contacts with PIP_2 are shown. The distance between residue V134 in the IDR C-terminus and the membrane midplane was either not restrained (top panels) or restrained at a specific height (5-9nm, bottom panels) to emulate the role of the ARD on IDR positioning.

For all conditions shown in (a-c), four replicate simulations per IDR sequence were carried out for ~38 µs and contact averages were calculated from the last ~28 µs of each simulation. Error bars depict the standard error of the mean (SEM) of the individual replicate simulations.
d Heat map showing the probability of two coarse-grained atoms of the IDR being in contact with each other in simulations of the native IDR (left), the patch mutant (center) and the IDR^{AAWAA} mutant (right). Contact maps are calculated from pooling the last ~28 µs of all four replicate simulations without height restraints acting on V134. A contact is counted if the coarse-grained atoms are closer than 0.6 nm to each other. Red boxes indicate the area of N-terminal patch interaction with the PBS. Of note, the absence of persistent long-range interactions between the N-terminal patch and the C-terminal PIP_2 binding site for the IDR in the MD simulations can be explained if the interactions are dominated by long-range electrostatics, which are comparably poor represented in the coarse-grained simulation model.
Source data are provided as a Source Data file.

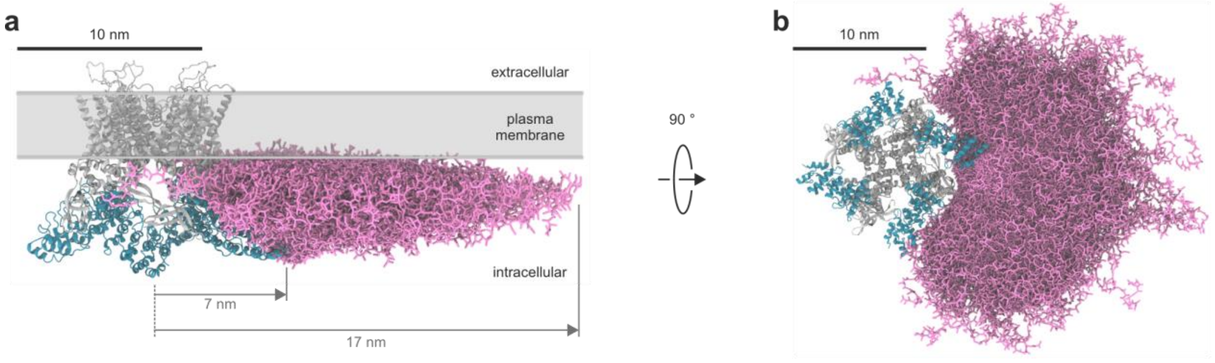

Supplementary Figure13: Structural model of the conformational space sampled by the IDR of a single TRPV4 subunit in the presence of the plasma membrane.
a, b Superimposed IDR conformations (pink licorice) from coarse-grained MD simulations. The transmembrane core (grey) of *G. gallus* TRPV4 including the ARDs (cyan) has been predicted by AlphaFold2[11].

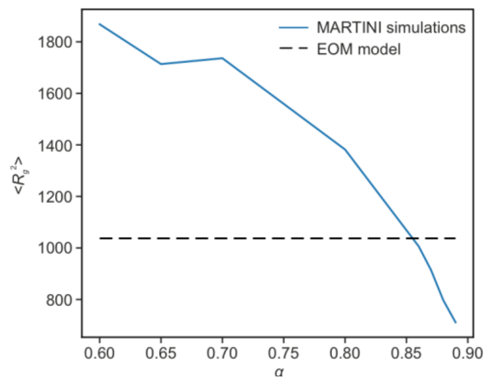

Supplementary Figure 14: Optimizing the scaling factor in MD simulations to best describe the measured R_g distribution of the native IDR.
Averaged squared R_g ($<R_g^2>$) in simulations with different rescaling constants of the protein/protein interactions (α). All simulations were performed without a membrane. The dotted black line shows the averaged squared R_g of the EOM refined model (Fig. 1, Supplementary Figure 3).
Source data are provided as a Source Data file.

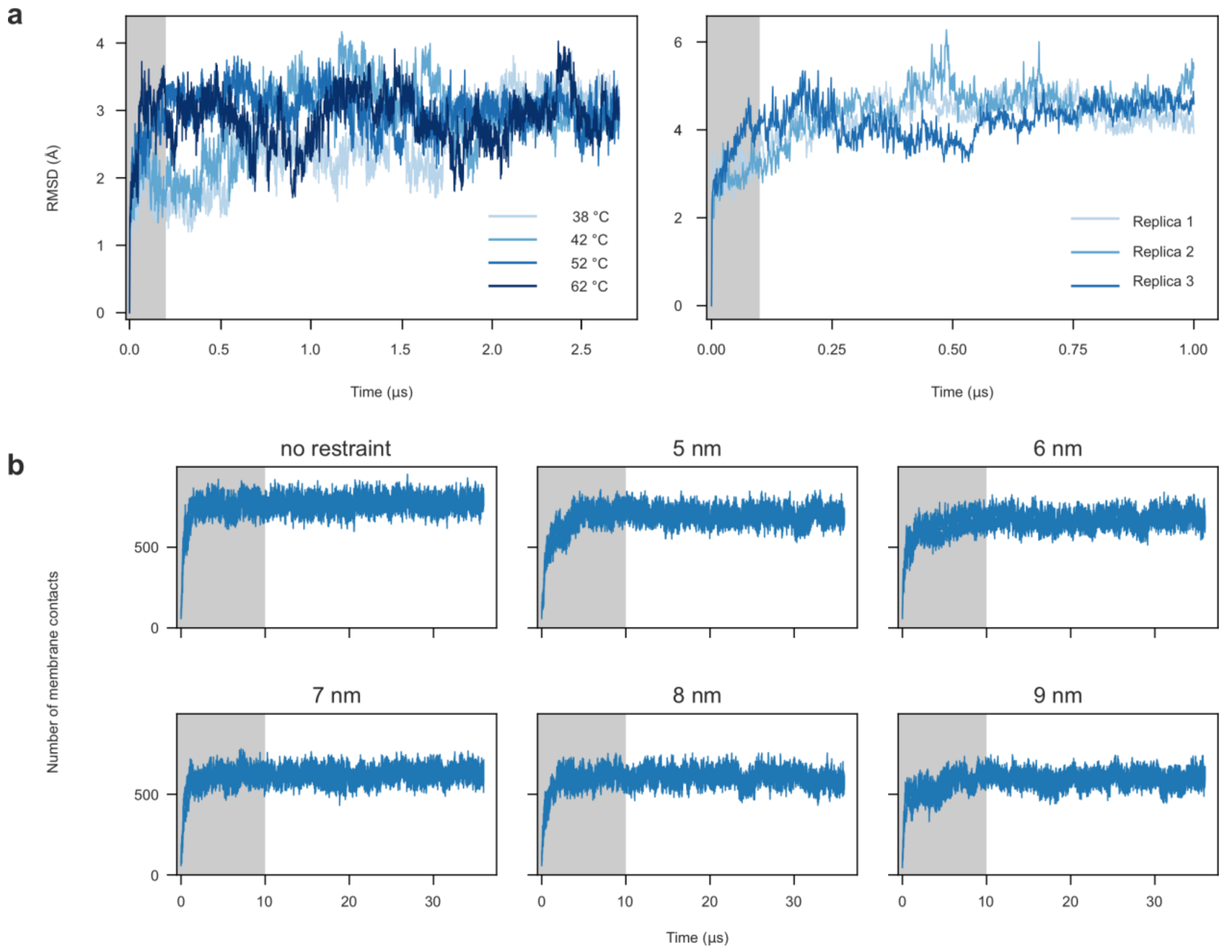

Supplementary Figure 15: Convergence of MD simulations.
a Root-mean-square-deviation (RMSD) with respect to the starting structure as function of time for the isolated ARD in solution at different temperatures (left) and for the full tetrameric TRPV4 core in a POPC lipid membrane (right). The grey shaded area (left) was excluded from all analyses.
b Number of contacts between the WT IDR and the membrane lipids as function of time averaged over 4 replicates per height restraint (blue line) with the SEM shown as blue shaded area. The first 10 µs of each simulation (grey shaded area) were excluded from all analyses.
Source data are provided as a Source Data file.

Supplementary Table 1 – SAXS data reporting table for native TRPV4 NTD, ARD and IDR as well as PIP_2-binding site mutants.

**Sample details**					
SAMPLE	NTD	NTD^{AAWAA}	ARD	IDR	IDR^{AAWAA}
SASBDB Accession Codes					
Organism			*Gallus gallus*		
NCBI protein accession ID			395427		
(amino acid range)	2-382	2-382	135-382	2-134	2-134
SEC-SAXS buffer			20 mM Tris pH 7.0, 10 mM DTT		
NaCl concentration		300 mM		100 mM	
Sample injection volume	40 µl	40 µl	40 µl	40 µl	40 µl
Sample injection conc.	10.0 mg/ml	8.2 mg/ml	10.7 mg/ml	10.1 mg/ml	12.9 mg/ml
SEC column		S200 Increase 5/150		S75 Increase 5/150	
SEC flow rate			0.3 ml/min		
SEC temperature			20 °C		
**Instrument details**					
Instrument			EMBL P12 bioSAXS beam line, DESY, Hamburg		
Exposure time/# frames			0.25 s (2400)		
X-ray wavelength/energy			0.124 nm (9996.5 eV)		
Sample-to-detector distance			3 m		
Scattering intensity scale			arb. units		
SEC-SAXS primary data processing			CHROMIXS (ATSAS 3.0.1)		
# frames used for averaging	104	95	79	59	60
Working s-range (nm^{-1})	0.07-3.70	0.06-4.20	0.09-4.98	0.08-5.00	0.10-5.60
**Guinier analysis:**					
Primary data analysis software			PRIMUS (ATSAS 3.0.1)		
Guinier $I(0)$ (σ)	1721(8)	5002(13)	3600(5)	1997(9)	4629(10)
R_g (Guinier, nm) (σ)	3.68(0.03)	4.02(0.01)	2.33(0.01)	3.20(0.02)	3.26(0.02)
sR_g range	0.26-1.20	0.40-1.01	0.21-1.30	0.25-1.30	0.35-1.29
**p(r) analysis:**					
Method			GNOM 5		
$I(0)$, POR (σ)	1754(11)	5147(17)	3644(9)	2009(11)	4714(12)
R_g (POR, nm) (σ)	4.06(0.07)	4.53(0.04)	2.49(0.02)	3.44(0.05)	3.50(0.02)
D_{max} (nm)	19.0	19.5	11.5	14.5	14.5
Quality of fit, CorMap p / χ^2	0.62/1.03	0.69/0.99	0.50/1.07	0.90/0.98	0.26/1.07
Porod volume (nm^3)	59	78	41	58	37
Shape classification	flexible	flexible	compact	random chain	random chain
**Molecular Weight analysis:**					
MW, calculated from amino acid sequence, kDa	42.5	42.2	28.0	14.5	14.2
MW from SAXS data, kDa	38-42	48-56	26-29	24-29	22-25
**Ab initio modelling:**					
Method			DAMMIN		
Symmetry			P1		
#models used for averaging			9		
Normalized Spatial Discrepancy, NSD			0.58		
Resolution estimate, nm			24		
Quality-of-fit, CorMap p / χ^2			0.45/1.06		
**Rigid body/Normal mode modelling:**					
Method			SREFLEX		
Symmetry			P1		
Template			PDB ID: 3W9G		
Initial template fit, CorMap p / χ^2			1.44e-43/3.33		
Final model fit, CorMap p / χ^2			4.05e-07/1.21		
**Ensemble modelling:**					
Method	EOM	EOM		EOM	EOM
Symmetry	P1	P1		P1	P1
Template	PDB ID: 3W9G	PDB ID: 3W9G		-	-
Final model fit, CorMap p / χ^2	0.72/0.99	0.52/0.99		0.97/0.98	0.19/1.3

Supplementary Table 2 – SAXS data reporting table for TRPV4 NTD deletion constructs.

**Sample details**				
SAMPLE	NTD$^{\Delta N120}$	NTD$^{\Delta N104}$	NTD$^{\Delta N97}$	NTD$^{\Delta N54}$
SASBDB Accession Codes				
Organism	*Gallus gallus*			
NCBI protein accession ID	395427			
(amino acid range)	121-382	105-382	98-382	55-134
SEC-SAXS buffer	20 mM Tris pH 7.0, 10 mM DTT			
NaCl concentration	300 mM			
Sample injection volume	40 µl	40 µl	40 µl	40 µl
Sample injection conc.	7.4 mg/ml	9.5 mg/ml	6.5 mg/ml	3.7 mg/ml
SEC column	S200 Increase 5/150			
SEC flow rate	0.3 ml/min			
SEC temperature	20 ^{o}C			
**Instrument details**				
Instrument	EMBL P12 bioSAXS beam line, DESY, Hamburg			
Exposure time/# frames	0.25 s (540)			
X-ray wavelength/energy	0.124 nm (9996.5 eV)			
Sample-to-detector distance	3 m			
Scattering intensity scale	arb. units			
SEC-SAXS primary data processing	CHROMIXS (ATSAS 3.0.1)			
# frames used for averaging	33	28	33	30
Working s-range (nm^{-1})	0.15-3.7	0.20-2.88	0.18-3.17	0.08-2.7
**Guinier analysis:**				
Primary data analysis software	PRIMUS (ATSAS 3.0.1)			
Guinier $I(0)$ (σ)	2046(4)	5364(7)	1828(3)	671(2)
R_g (Guinier, nm) (σ)	2.67(0.01)	2.68(0.01)	2.52(0.01)	2.63(0.01)
sR_g range	0.42-1.15	0.67-1.24	0.44-1.33	0.21-1.30
**p(r) analysis:**				
Method	GNOM 5			
$I(0)$, POR (σ)	2074(5)	5574(9)	1868(5)	673(2)
R_g (POR, nm) (σ)	2.86(0.02)	3.01(0.01)	2.76(0.02)	2.68(0.02)
D_{max} (nm)	12.5	13.0	11.9	9.5
Quality of fit, CorMap p / χ^2	0.71/1.09	0.06/1.12	0.29/1.05	0.36/1.00
Porod volume (nm^3)	43	46	42	64
Shape classification	extended	extended	compact	flat
**Molecular Weight analysis:**				
MW, calculated from amino acid sequence, kDa	29.4	31.4	32.3	37.0
MW from SAXS data, kDa	37-40	25-29	31-35	29-31
**Ensemble modeling**				
Method	EOM	EOM	EOM	EOM
Symmetry	P1	P1	P1	P1
Template	PDB ID: 3W9G	PDB ID: 3W9G	PDB ID: 3W9G	PDB ID: 3W9G
Final model fit, CorMap p / χ^2	1.13e-64/39.45	3.01e-66/14.67	7.16e-04/1.14	0.01/1.02

Supplementary Table 3 – Overview of data obtained by hydrogen/deuterium exchange mass spectrometry (HDX-MS)

Protein	IDR	ARD	NTD
Conditions of H/D exchange	25 °C in 20 mM Tris-Cl pH 7.0, 300 mM NaCl; final D_2O = 90%		
Time course of H/D exchange	10/30/100/1,000/10,000 s		
Replicates	3 independent protein preparations measured with 3 technical replicates (separate H/D exchange reactions) each.		
Number of Peptides	17	93	110
Average peptide length (aa)	16.12	11.83	12.49
Sequence coverage (%)	64.7	100	87.7
Redundancy	3.19	4.44	4.11
Back-exchange	No correction for back-exchange based on a fully deuterated sample conducted.		
Repeatability (average SD)	0.054 Da / 0.437%	0.070 Da / 0.700%	0.066 Da / 0.676%
Significance criterium applied	5% difference in relative and 0.5 Da difference in absolute (Houde et al., 2011)[12] HDX		

Supplementary Table 4 – Lipid composition of the plasma membrane inner leaflet used in all molecular dynamics simulations.

Lipid type	MARTINI model	Charge	Abundance [%]
Cholesterol	CHOL	0	20.03
1-palmitoyl-2-oleoyl-glycero-3-phosphocholine	POPC	0	69.14
1,2-dioleoyl-glycero-3-phosphoserine	DOPS	-1	9.94
1-palmitoyl-2-linoleoyl-*sn*-glycero-3-phosphoinositol-4,5-bisphosphate	POP2	-5	0.89

Supplementary Table 5 – Overview over performed MD simulations. All simulations were performed with 150mM NaCl.

System	Type	Replicates	Simulated time min; max of replicates (µs)	Box dimensions (nm^3)	No. atoms/particles	No. water molecules	Lipid type/no. molecules
isolated ARD	atomistic	4 (38, 42, 52, 62 ℃)	2.71; 2.86	10 x 10 x 7.1 (rhombic dodecahedron)	91315	21801	n.a.
TRPV4 core	atomistic	3	1	19.5 x 19.5 x 16.3	629980	149974	POPC/1029
IDR no restraint	CG	4	37.7; 40.8	19.4 x 19.4 x 20.4	66853	49781	POPC/932; CHOL/270; DOPS/134; PIP2/12
IDR restraint 5nm	CG	4	36.0; 40.0	19.4 x 19.4 x 20.4	66853	49781	POPC/932; CHOL/270; DOPS/134; PIP2/12
IDR restraint 6nm	CG	4	39.4; 40.3	19.4 x 19.4 x 20.4	66853	49781	POPC/932; CHOL/270; DOPS/134; PIP2/12
IDR restraint 7nm	CG	4	38.1; 39.7	19.4 x 19.4 x 20.4	66853	49781	POPC/932; CHOL/270; DOPS/134; PIP2/12
IDR restraint 8nm	CG	4	38.8; 41.4	19.4 x 19.4 x 20.4	66853	49781	POPC/932; CHOL/270; DOPS/134; PIP2/12
IDR restraint 9nm	CG	4	37.9; 41.5	19.4 x 19.4 x 20.4	66853	49781	POPC/932; CHOL/270; DOPS/134; PIP2/12
IDR^{AAWAA} no restraint	CG	4	38.0; 39.7	19.4 x 19.4 x 20.4	66858	49803	POPC/932; CHOL/270; DOPS/134; PIP2/12
IDR^{AAWAA} restraint 5nm	CG	4	35.9; 39.4	19.4 x 19.4 x 20.4	66858	49803	POPC/932; CHOL/270; DOPS/134; PIP2/12
IDR^{AAWAA} restraint 6nm	CG	4	38.0; 39.4	19.4 x 19.4 x 20.4	66858	49803	POPC/932; CHOL/270; DOPS/134; PIP2/12
IDR^{AAWAA} restraint 7nm	CG	4	35.0; 41.2	19.4 x 19.4 x 20.4	66858	49803	POPC/932; CHOL/270; DOPS/134; PIP2/12
IDR^{AAWAA} restraint 8nm	CG	4	38.6; 40.7	19.4 x 19.4 x 20.4	66858	49803	POPC/932; CHOL/270; DOPS/134; PIP2/12
IDR^{AAWAA} restraint 9nm	CG	4	39.1; 39.8	19.4 x 19.4 x 20.4	66858	49803	POPC/932; CHOL/270; DOPS/134; PIP2/12
IDR^{Patch} no restraint	CG	4	36.3; 41.0	19.4 x 19.4 x 20.4	66871	49798	POPC/932; CHOL/270; DOPS/134; PIP2/12
IDR^{Patch} restraint 5nm	CG	4	35.6; 41.4	19.4 x 19.4 x 20.4	66871	49798	POPC/932; CHOL/270; DOPS/134; PIP2/12
IDR^{Patch} restraint 6nm	CG	4	37.4; 41.1	19.4 x 19.4 x 20.4	66871	49798	POPC/932; CHOL/270; DOPS/134; PIP2/12
IDR^{Patch} restraint 7nm	CG	4	35.4; 40.9	19.4 x 19.4 x 20.4	66871	49798	POPC/932; CHOL/270; DOPS/134; PIP2/12
IDR^{Patch} restraint 8nm	CG	4	37.4; 42.6	19.4 x 19.4 x 20.4	66871	49798	POPC/932; CHOL/270; DOPS/134; PIP2/12
IDR^{Patch} restraint 9nm	CG	4	35.1; 41.8	19.4 x 19.4 x 20.4	66871	49798	POPC/932; CHOL/270; DOPS/134; PIP2/12

Supplementary References

1. Uversky, V. N. Size-Exclusion Chromatography in Structural Analysis of Intrinsically Disordered Proteins. in *Intrinsically Disordered Protein Analysis: Volume 2, Methods and Experimental Tools* (eds. Uversky, V. N. & Dunker, A. K.) 179–194 (Springer, 2012). doi:10.1007/978-1-4614-3704-8_11.
2. Goretzki, B., Tebbe, F., Mitrovic, S.-A. & Hellmich, U. A. Backbone NMR assignments of the extensive human and chicken TRPV4 N-terminal intrinsically disordered regions as important players in ion channel regulation. *Biomol NMR Assign* **16**, 205–212 (2022).
3. Franke, D. *et al.* ATSAS 2.8: a comprehensive data analysis suite for small-angle scattering from macromolecular solutions. *J Appl Cryst* **50**, 1212–1225 (2017).
4. Franke, D., Jeffries, C. M. & Svergun, D. I. Correlation Map, a goodness-of-fit test for one-dimensional X-ray scattering spectra. *Nat Methods* **12**, 419–422 (2015).
5. Pearson, K. X. On the criterion that a given system of deviations from the probable in the case of a correlated system of variables is such that it can be reasonably supposed to have arisen from random sampling. *The London, Edinburgh, and Dublin Philosophical Magazine and Journal of Science* **50**, 157–175 (1900).
6. Micsonai, A. *et al.* BeStSel: a web server for accurate protein secondary structure prediction and fold recognition from the circular dichroism spectra. *Nucleic Acids Research* **46**, W315–W322 (2018).
7. Bernadó, P., Mylonas, E., Petoukhov, M. V., Blackledge, M. & Svergun, D. I. Structural Characterization of Flexible Proteins Using Small-Angle X-ray Scattering. *J. Am. Chem. Soc.* **129**, 5656–5664 (2007).
8. McWilliam, H. *et al.* Analysis Tool Web Services from the EMBL-EBI. *Nucleic Acids Research* **41**, W597–W600 (2013).
9. Ashkenazy, H. *et al.* ConSurf 2016: an improved methodology to estimate and visualize evolutionary conservation in macromolecules. *Nucleic Acids Research* **44**, W344–W350 (2016).
10. Schneider, C. A., Rasband, W. S. & Eliceiri, K. W. NIH Image to ImageJ: 25 years of image analysis. *Nat Methods* **9**, 671–675 (2012).
11. Jumper, J. *et al.* Highly accurate protein structure prediction with AlphaFold. *Nature* **596**, 583–589 (2021).
12. Houde, D., Berkowitz, S. A. & Engen, J. R. The utility of hydrogen/deuterium exchange mass spectrometry in biopharmaceutical comparability studies. *J Pharm Sci* **100**, 2071–2086 (2011).
