# Supplementary material for: Crosstalk between regulatory elements in disordered TRPV4 N-terminus modulates lipid-dependent channel activity
Source: Nat Commun. 2023 Jul 13;14:4165. doi: 10.1038/s41467-023-39808-4 (PMC10344929; doi:10.1038/s41467-023-39808-4)
Supplement: Supplementary file 3 — Description of Additional Supplementary Files [file 41467_2023_39808_MOESM3_ESM.pdf]

Description of Additional Supplementary Files

Supplementary Data 1: Summary of HDX-MS analyses and full list of the peptides obtained for different TRPV4 protein constructs.
Supplementary Data 2: Peptides analyzed by HDX-MS mapped on TRPV4-NTD sequence.
Supplementary Data 3: Summary of XL-MS analyses

Supplementary Movie 1: Atomistic MD simulation of core *G. gallus* TRPV4 ion channel in a lipid membrane
Supplementary Movie 2: Ensemble of TRPV4 IDR structures from MARTINI Simulations on the membrane, side view.
Supplementary Movie 3: Ensemble of TRPV4 IDR structures from MARTINI Simulations on the membrane, view from the cytosol.
