# Supplementary figures and images for: Crosstalk between regulatory elements in disordered TRPV4 N-terminus modulates lipid-dependent channel activity
Source: Nat Commun. 2023 Jul 13;14:4165. doi: 10.1038/s41467-023-39808-4 (PMC10344929; doi:10.1038/s41467-023-39808-4)

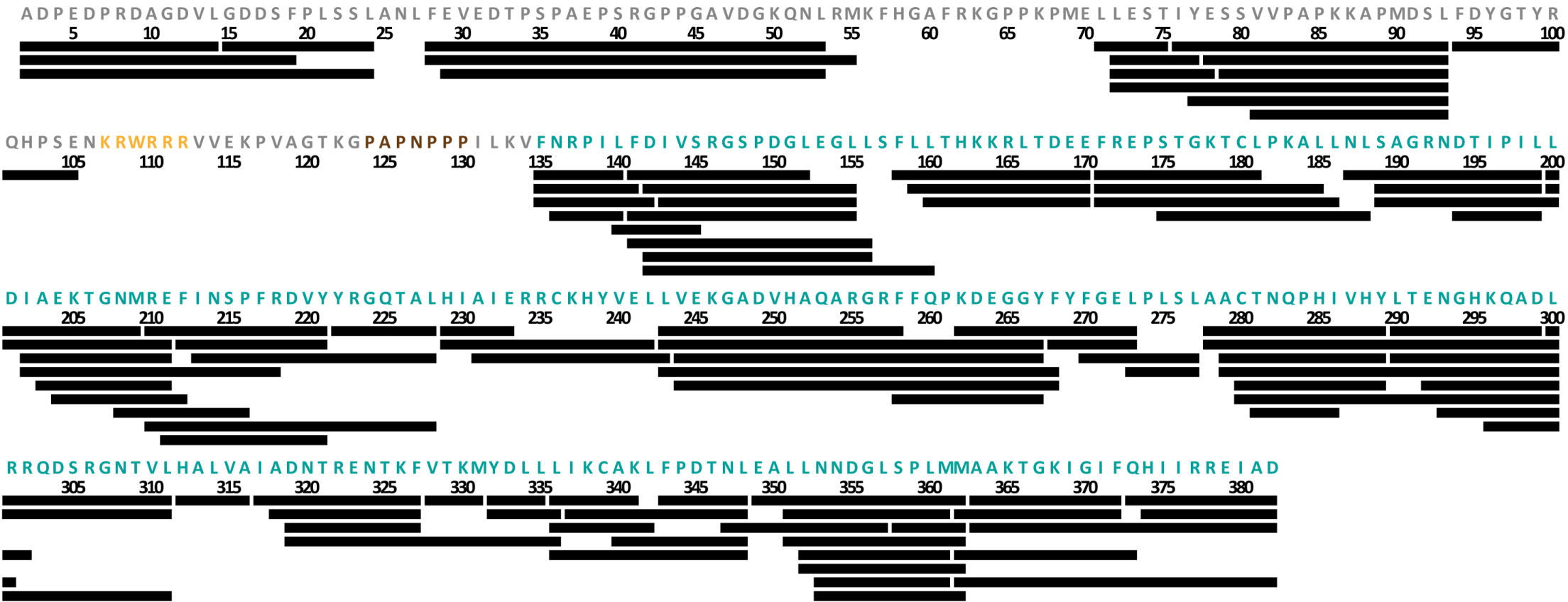

Supplement: Supplementary file 5 — Supplementary Data 2 [file 41467_2023_39808_MOESM5_ESM.pdf]
