# Supplementary material for: Crosstalk between regulatory elements in disordered TRPV4 N-terminus modulates lipid-dependent channel activity
Source: Nat Commun. 2023 Jul 13;14:4165. doi: 10.1038/s41467-023-39808-4 (PMC10344929; doi:10.1038/s41467-023-39808-4)
Supplement: Supplementary file 10 — Reporting Summary [file 41467_2023_39808_MOESM10_ESM.pdf]

nature portfolio

Corresponding author(s): Ute A. Hellmich

Last updated by author(s): 2023/09/06

Reporting Summary

Nature Portfolio wishes to improve the reproducibility of the work that we publish. This form provides structure for consistency and transparency in reporting. For further information on Nature Portfolio policies, see our Editorial Policies and the Editorial Policy Checklist.

Statistics

For all statistical analyses, confirm that the following items are present in the figure legend, table legend, main text, or Methods section.

n/a	Confirmed	
☐	☒	The exact sample size (*n*) for each experimental group/condition, given as a discrete number and unit of measurement
☐	☒	A statement on whether measurements were taken from distinct samples or whether the same sample was measured repeatedly
☐	☒	The statistical test(s) used AND whether they are one- or two-sided<br>*Only common tests should be described solely by name; describe more complex techniques in the Methods section.*
☒	☐	A description of all covariates tested
☒	☐	A description of any assumptions or corrections, such as tests of normality and adjustment for multiple comparisons
☐	☒	A full description of the statistical parameters including central tendency (e.g. means) or other basic estimates (e.g. regression coefficient) AND variation (e.g. standard deviation) or associated estimates of uncertainty (e.g. confidence intervals)
☐	☒	For null hypothesis testing, the test statistic (e.g. *F*, *t*, *r*) with confidence intervals, effect sizes, degrees of freedom and *P* value noted<br>*Give P values as exact values whenever suitable.*
☒	☐	For Bayesian analysis, information on the choice of priors and Markov chain Monte Carlo settings
☒	☐	For hierarchical and complex designs, identification of the appropriate level for tests and full reporting of outcomes
☒	☐	Estimates of effect sizes (e.g. Cohen's *d*, Pearson's *r*), indicating how they were calculated

Our web collection on statistics for biologists contains articles on many of the points above.

Software and code

Policy information about availability of computer code

Data collection	SAXS: Automated sample injection and data collection were controlled using the BECQUEREL beam line control software. HDX-MS data were collected with MassLynx MS version 4.1 (Waters). Molecular dynamics trajectories were simulated using Gromacs 2020.3. Ca-imaging: Ratiometric imaging data were collected using AxioVision software (Zeiss, v4).
Data analysis	Freely available computational tools were used to investigate the properties of N-terminal TRPV4 constructs. Sequence conservation was determined with ConSurf (https://consurf.tau.ac.il/consurf_index.php. Overall charge (z) and charge distribution of IDR deletion constructs were determined with ProtPi (www.protpi.ch and www.bioinformatics.nl/cgi-bin/emboss/charge). Gel densitometry analysis was carried out with ImageJ. The IDR charge gradient was plotted with the PepCalc tool (https://pepcalc.com/).<br>SAXS: 2D-to-1D data reduction was performed using the SASFLOW pipeline incorporating RADAVER from the ATSAS 2.8 and ATSAS 3.0.1 suite of software tools. Individual frames obtained for each SEC-SAXS run were processed using CHROMIXS. SAXS data analysis was performed using PRIMUS within ATSAS3.0.1. DAMMIN and SREFLEX were used for modelling. (All available via https://www.embl-hamburg.de/biosaxs/software.html)<br>MS: Peptides were identified and deuterium incorporation evaluated with the ProteinLynx Global SERVER 3.0.1 (PLGS) and DynamX 3.0 (Waters). Crosslink analysis was done with the xQuest/xProphet pipeline.<br>SEC -MALS: analysis was done with ASTRA 7 Software (Wyatt Technology).<br>NMR: Processing was done with TOPSIN3.6 (Bruker).<br>MD: To analyze molecular dynamics trajectories, custom Python3.9 code was written and deposited in a zenodo repository along with raw trajectory data (doi:10.5281/zenodo.7957940)

For manuscripts utilizing custom algorithms or software that are central to the research but not yet described in published literature, software must be made available to editors and reviewers. We strongly encourage code deposition in a community repository (e.g. GitHub). See the Nature Portfolio guidelines for submitting code & software for further information.

Data

Policy information about availability of data

All manuscripts must include a data availability statement. This statement should provide the following information, where applicable:

- Accession codes, unique identifiers, or web links for publicly available datasets
- A description of any restrictions on data availability
- For clinical datasets or third party data, please ensure that the statement adheres to our policy

The NMR backbone assignment of the G. gallus TRPV4 N-terminal intrinsically disordered region has been deposited in the BioMagResBank (www.bmrb.io) under the accession number 51172 [https://dx.doi.org/10.13018/BMR51172]. The XL-MS and HDX-MS data have been deposited to the ProteomeXchange Consortium via the PRIDE partner repository92 with the project accession numbers PXD038153 [https://proteomecentral.proteomexchange.org/cgi/GetDataset?ID=PXD038153] and PXD041067 [https://proteomecentral.proteomexchange.org/cgi/GetDataset?ID=PXD041067], respectively. A spreadsheet with a summary of the conditions used for HDX-MS analyses and a full list of the peptides obtained for different TRPV4 protein constructs is available in Supplemental Dataset 1. The SAXS data have been deposited in the SASBDB under the accession numbers SASDQE8 [https://www.sasbdb.org/data/SASDQE8/] (ARD), SASDQF8 [https://www.sasbdb.org/data/SASDQF8/] (NTD), SASDQG8 [https://www.sasbdb.org/data/SASDQG8/] (NTDAAWAA), SASDQH8 [https://www.sasbdb.org/data/SASDQH8/] (NTDΔN54), SASDQJ8 [https://www.sasbdb.org/data/SASDQJ8/] (NTDΔN97), SASDQK8 [https://www.sasbdb.org/data/SASDQK8/] (NTDΔN104), SASDQL8 [https://www.sasbdb.org/data/SASDQL8/] (NTDΔN120), SASDQM8 [https://www.sasbdb.org/data/SASDQM8/] (IDR), SASDQN8 [https://www.sasbdb.org/data/SASDQN8/] (IDRAAWAA). Source data are provided with this paper. The high-resolution protein structures of the G. gallus TRPV4 ankyrin repeat domain used in this study is available in the protein database under the accession code 3W9G [http://doi.org/10.2210/pdb3W9G/pdb].

Human research participants

Policy information about studies involving human research participants and Sex and Gender in Research.

Reporting on sex and gender	Not relevant to this study.
Population characteristics	Not relevant to this study.
Recruitment	Not relevant to this study.
Ethics oversight	Not relevant to this study.

Note that full information on the approval of the study protocol must also be provided in the manuscript.

Field-specific reporting

Please select the one below that is the best fit for your research. If you are not sure, read the appropriate sections before making your selection.

[x] Life sciences [ ] Behavioural & social sciences [ ] Ecological, evolutionary & environmental sciences

For a reference copy of the document with all sections, see nature.com/documents/nr-reporting-summary-flat.pdf

Life sciences study design

All studies must disclose on these points even when the disclosure is negative.

Sample size	Sample sizes are reported in the material and methods section and adhere to the respective standards of the field. All measurements were carried out at least in biological duplicates or technical triplicates (Trp fluorescence). For ratiometric calcium imaging, sample size was predetermined to be at least n=6 from two experimental replicates obtained from three independent experiments on different days. This predetermination was based on results from prior unrelated experiments demonstrating that this sample size yielded standard error of the mean that was less than 10% of the mean.<br><br>For HDX-MS, three technical replicates of three individual protein preparations (total of 9 replicates per deuteration time point) were analyzed exceeding the minimal criteria for HDX-MS experiments outlined in the manuscript by Masson GR et al. (2019) Nat Methods, which describes common standards for HDX-MS experiments.
Data exclusions	No data exclusions with the exception of one MD simulation (from n=4) as detailed in the material and method section, since the protein reached over the periodic boundary to the other face of the membrane. This exclusion was not preestablished.<br>In HDX-MS, some peptides were omitted from further analysis in case of low signal-to-noise ratio or spectral overlaps. This exclusion was not preestablished.

Replication	Biophysical measurements were carried out at least in biological duplicates or technical triplicates (Trp fluorescence), all replicates were included in the statistical analyses. All experiments were reproducible.
Randomization	Not applicable to the biophysical experiments due to the organization of the wet lab work flow. Samples were measured when they became available (upon purifictation) and were treated identically with regard to storage conditions. For ratiometric calcium imaging, the center of each coverslip was imaged. For live imaging experiments, the experimental conditions were recorded in an alternating fashion on a given day, and the order was then changed on subsequent days.
Blinding	Experimentalists were not blinded to sample identity as it was deemed not feasible within the wet lab work flow. In many cases, data analyses required sample knowledge (NMR assignments, HDX-MS).<br>However, data analysis was carried out in a blinded fashion where possible, i.e. without sharing prior hypotheses and by transferring data sets to researchers not involved in data collection. Blinding for calcium imaging was deemed unnecessary as generation of cell ROIs and subsequent quantification was performed in a standardized manner with most steps being automated.

Reporting for specific materials, systems and methods

We require information from authors about some types of materials, experimental systems and methods used in many studies. Here, indicate whether each material, system or method listed is relevant to your study. If you are not sure if a list item applies to your research, read the appropriate section before selecting a response.

Materials & experimental systems

n/a	Involved in the study
☐	☒ Antibodies
☐	☒ Eukaryotic cell lines
☒	☐ Palaeontology and archaeology
☒	☐ Animals and other organisms
☒	☐ Clinical data
☒	☐ Dual use research of concern

Methods

n/a	Involved in the study
☒	☐ ChIP-seq
☒	☐ Flow cytometry
☒	☐ MRI-based neuroimaging

Antibodies

Antibodies used	rabbit anti-GFP (Thermo Fisher Scientific, A-11122), 391 rabbit anti-β-actin (Cell Signaling Technology, 4967) and HRP-conjugated monoclonal mouse anti-392 rabbit IgG, light chain specific (Jackson ImmunoResearch, 211-032-171). All antibodies were used at 1:1000.
Validation	All antibodies have been validated by the companies and are widely used in the literature. Specific information can be obtained from the respective websites using the product numbers referenced above.<br><br>Actin (information from the cell signaling website): Examination of several cell lines and/or tissues of known expression levels allows accurate determination of species cross-reactivity and verifies specificity. The use of siRNA transfection or knockout cell lines verifies target specificity.<br>Side-by-side comparison of lots to ensure lot-to-lot consistency. Optimal dilutions and buffers are predetermined, positive and negative cell extracts are specified, and detailed protocols are already optimized, saving valuable time and reagents.<br>GFP: This antibody has been used in over 1,600 papers as listed on the product website (https://www.thermofisher.com/antibody/product/GFP-Antibody-Polyclonal/A-11122). The antibody to GFP was further validated by our lab by western blot and immunofluorescence using untransfected and epitope-tag transfected cells as negative and positive controls, respectively.<br>HRP conjugated secondary: From manufacturer: The antibody has been tested by ELISA to ensure minimal cross-reaction with bovine, goat, armenian hamster, horse, human, mouse, rat and sheep immunoglobulins, but it may cross-react with immunoglobulins from other species.

Eukaryotic cell lines

Policy information about cell lines and Sex and Gender in Research

Cell line source(s)	MN-1 cells were obtained from Dr. Kenneth Fischbeck at NIH. There is no commercial source for MN-1 cells.
Authentication	Cells were not authenticated but have been widely used in the literature for the described experiments.
Mycoplasma contamination	Cell lines have not been tested for mycoplasma
Commonly misidentified lines (See ICLAC register)	No commonly misidentified cell line was used in the study.
